# Inferring shape transformations in a drawing task

**DOI:** 10.3758/s13421-023-01452-0

**Published:** 2023-09-05

**Authors:** Filipp Schmidt, Henning Tiedemann, Roland W. Fleming, Yaniv Morgenstern

**Affiliations:** 1 Department of Experimental Psychology, Justus Liebig University Giessen, Otto-Behaghel-Str. 10F, 35394 Giessen, Germany; 2 Center for Mind, Brain and Behavior (CMBB), University of Marburg and Justus Liebig University, Giessen, Germany; 3 University of Leuven (KU Leuven), Leuven, Belgium

**Keywords:** Vision, Shape perception, Transformations, Drawing, Imagery

## Abstract

Many objects and materials in our environment are subject to transformations that alter their shape. For example, branches bend in the wind, ice melts, and paper crumples. Still, we recognize objects and materials across these changes, suggesting we can distinguish an object’s original features from those caused by the transformations (“shape scission”). Yet, if we truly understand transformations, we should not only be able to identify their signatures but also actively apply the transformations to new objects (i.e., through imagination or mental simulation). Here, we investigated this ability using a drawing task. On a tablet computer, participants viewed a sample contour and its transformed version, and were asked to apply the same transformation to a test contour by drawing what the transformed test shape should look like. Thus, they had to (i) infer the transformation from the shape differences, (ii) envisage its application to the test shape, and (iii) draw the result. Our findings show that drawings were more similar to the ground truth transformed test shape than to the original test shape—demonstrating the inference and reproduction of transformations from observation. However, this was only observed for relatively simple shapes. The ability was also modulated by transformation type and magnitude but not by the similarity between sample and test shapes. Together, our findings suggest that we can distinguish between representations of original object shapes and their transformations, and can use visual imagery to mentally apply nonrigid transformations to observed objects, showing how we not only perceive but also ‘understand’ shape.

**Supplementary Information:**

The online version contains supplementary material available at 10.3758/s13421-023-01452-0.

All objects and materials in our environment are a product of generative processes, such as growth or self-organization in response to physical forces. Many of these processes we experience as transformations of the shape of objects or their materials. For example, new shapes emerge when plants bud and flourish, when animals repose their limbs, when a towel is dropped in a pile, or when a child traces patterns in sand. These shape changes are a complex challenge to our visual and cognitive systems, which have to solve two complementary and linked inferences: recognizing objects across transformations (Biederman, 1987; DiCarlo et al., 2012; Logothetis & Sheinberg, 1996; Pasupathy et al., 2018; Riesenhuber & Poggio, 2000), and recognizing transformations across objects (Arnheim, 1974; Chen et al., 2021; Leyton, 1989; Ons & Wagemans, 2012; Pinna, 2010; Pinna & Deiana, 2015; Schmidt & Fleming, 2018; Spröte & Fleming, 2016).

Of these two inferences, much less is known about the recognition of transformations themselves, which has typically been tested implicitly. For example, previous work showed that only transformations that are interpreted as meaningful affect our visual perception. We only perceive illusory (apparent) motion when the intrusion of one object into another produces a physically plausible indentation (Chen & Scholl, 2016) and when motion trajectories of objects are in line with biomechanical (Kourtzi & Shiffrar, 1999) or material constraints (Kourtzi & Shiffrar, 2001). Moreover, judgements about the main axis and the front and back of objects are affected by whether their features are interpreted as belonging to the object or as a result of meaningful external transformations like a “bite” (Spröte et al., 2016). On the other hand, explicit tests of transformation recognition examined observers’ interpretations of particular changes in object shape to specific transformations. For example, observers can infer complex “happenings” from line drawings of simple squares (Pinna, 2010), growth or ageing from changes in body proportions or head shape (Mark & Todd, 1985; Pittenger & Todd, 1983; Schmidt & Fleming, 2016), and transformations like bleaching, burning, decaying, folding, bending, crumpling, or twisting from photographs of real objects (Schmidt & Fleming, 2018; Toscani et al., 2020; Yoonessi & Zaidi, 2010).

How do humans make such transformation inferences? On the one hand, there are definite limits to the ability to infer transformations (e.g., if they do not leave traces in the shape of the object or material; Leyton, 1989; Schmidt & Fleming, 2018). However, on the other hand, recent evidence suggests that observers can distinguish between shape features that “belong” to the object and those that “belong” to the transformation applied to the object, which allows them to distinguish between the object and the effects of the transformation (“shape scission”; Fleming & Schmidt, 2019; Phillips & Fleming, 2020; Schmidt et al., 2019; Spröte & Fleming, 2016). This is also reflected in distinct neural representations of features that preserve the identity of visual objects, and other, non-identity-preserving features (Henderson & Serences, 2019; Mocz et al., 2021; Ward et al., 2018; Xu and Vaziri-Pashkam, 2022). Consequently, observers are often able to isolate the original shape of an object from its transformations and—by comparing the transformation features to previously learned feature meanings—identify particular transformations.

Here, we test the extent to which participants can explicitly estimate transformations and mentally simulate the consequences of applying those transformations to other shapes. Previous studies showed that observers can to some extent match the amount of common transformations (e.g., bending) across objects, as well as discount the effects of the transformation for single objects (Spröte & Fleming, 2016). Using an unconstrained drawing task, we examine more challenging unfamiliar transformations. Specifically, we test whether observers can isolate relevant features—by comparing an object before and after a transformation—to an extent that allows them to “apply” the transformation to a different object by drawing the envisaged result.

In making such inferences, a number of factors are likely to be important. These include semantics, transformation type and magnitude, and shape similarity. Here, we aimed to minimize biases from semantic concepts and knowledge by using unfamiliar objects and transformations. Knowledge about particular transformations may bias drawings into the direction of a prototypical (e.g., “bending”) transformation. Knowledge about particular objects may bias drawings towards end states considered possible for those objects (e.g., in line with biomechanical plausibility; Heptulla Chatterjee et al., 1996). To allow for meaningful inferences, we show objects before and after transformation (in contrast to some previous studies that only showed a single, transformed object; e.g., Arnheim, 1974; Leyton, 1989; Pinna, 2010; Pittenger & Todd, 1983; Schmidt & Fleming, 2018).

Rather than on semantics, we focus on the role of transformation type, of transformation complexity and magnitude, and of the shape similarity between two objects. We expected that the ability to infer and reproduce transformations from looking at object shapes before and after transformation would be affected by all of these factors. For example, we hypothesized that the type of transformation would be important: It should be easier to mentally “fold” a towel the same way as another towel than to “crumple” it the same way (Fig. 1A). Also, transformation complexity should affect the difficulty of inferences and reproduction: It should be easier to mentally fold a rectangular sheet of paper into a triangle than to mentally fold it into a crane (Fig. 1B). Finally, we expected the magnitude of transformation and the similarity between the transformed object and the object to be transformed to be important. Specifically, it should be easier to identify a “strong” transformation (which, for example, produces a visible fold instead of a wrinkle); and it should be more difficult to reproduce transformation of an oblong towel (Fig. 1A) with square paper (Fig. 1B) than if both were oblong or square.

**Fig. 1:**
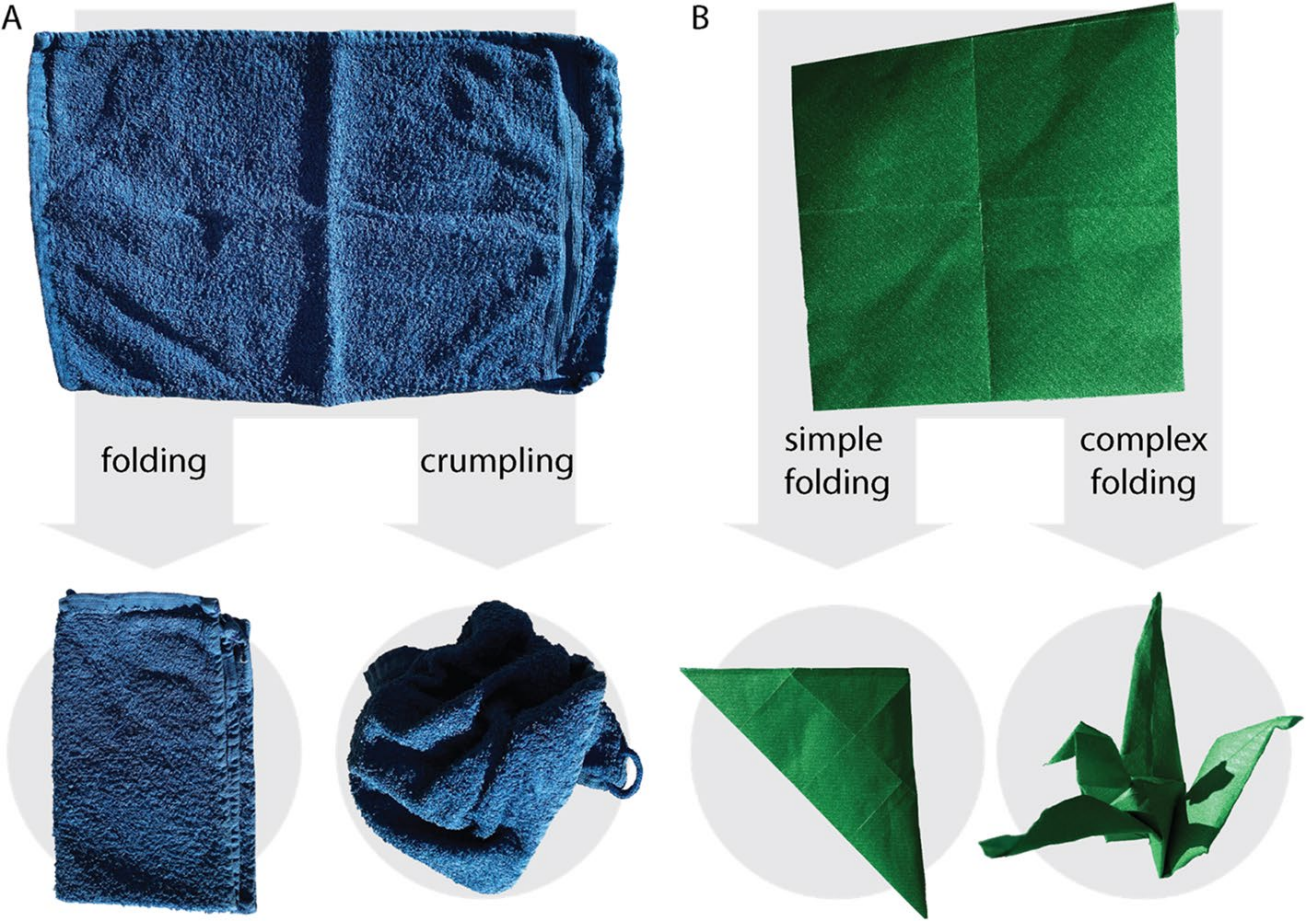
Examples of different transformed objects. **A** Towel, reshaped by a folding or crumpling transformation. **B** Sheet of paper, reshaped by different levels of transformation magnitude (simple or complex)

Consequently, we use a free-hand drawing task in which we present observers with a sample object before and after transformation, and a test object that they should apply the transformation to, and vary the (1) type of transformation, (2) magnitude of transformation, and (3) level of similarity between sample and test object. Also, we use a set of rather simple versus more complex contour stimuli.

## Method

### Transparency and openness

All data and stimuli are available at 10.5281/zenodo.8297790. Data were analyzed using MATLAB 2018a (Version 9.4.0; The MathWorks, Natick, MA). This study’s design and its analysis were not preregistered.

### Participants

Experiments were approved by the Local Ethics Committee of the Department of Psychology and Sports Sciences of the Justus Liebig University Giessen (LEK-2017-0046). Participants were recruited via a university mailing list or personal contact, calling specifically for people with good drawing skills. Participants in all experiments had normal or corrected-to-normal vision and were naïve to the purpose of the study. They gave informed consent and were treated in accordance with the ethical guidelines of the American Psychological Association (APA). Participants received financial compensation or course credits for their participation. The experiment with simple shapes was completed by 11 and that with complex shapes by 20 participants.

### Stimulus creation

**Fig. 2:**
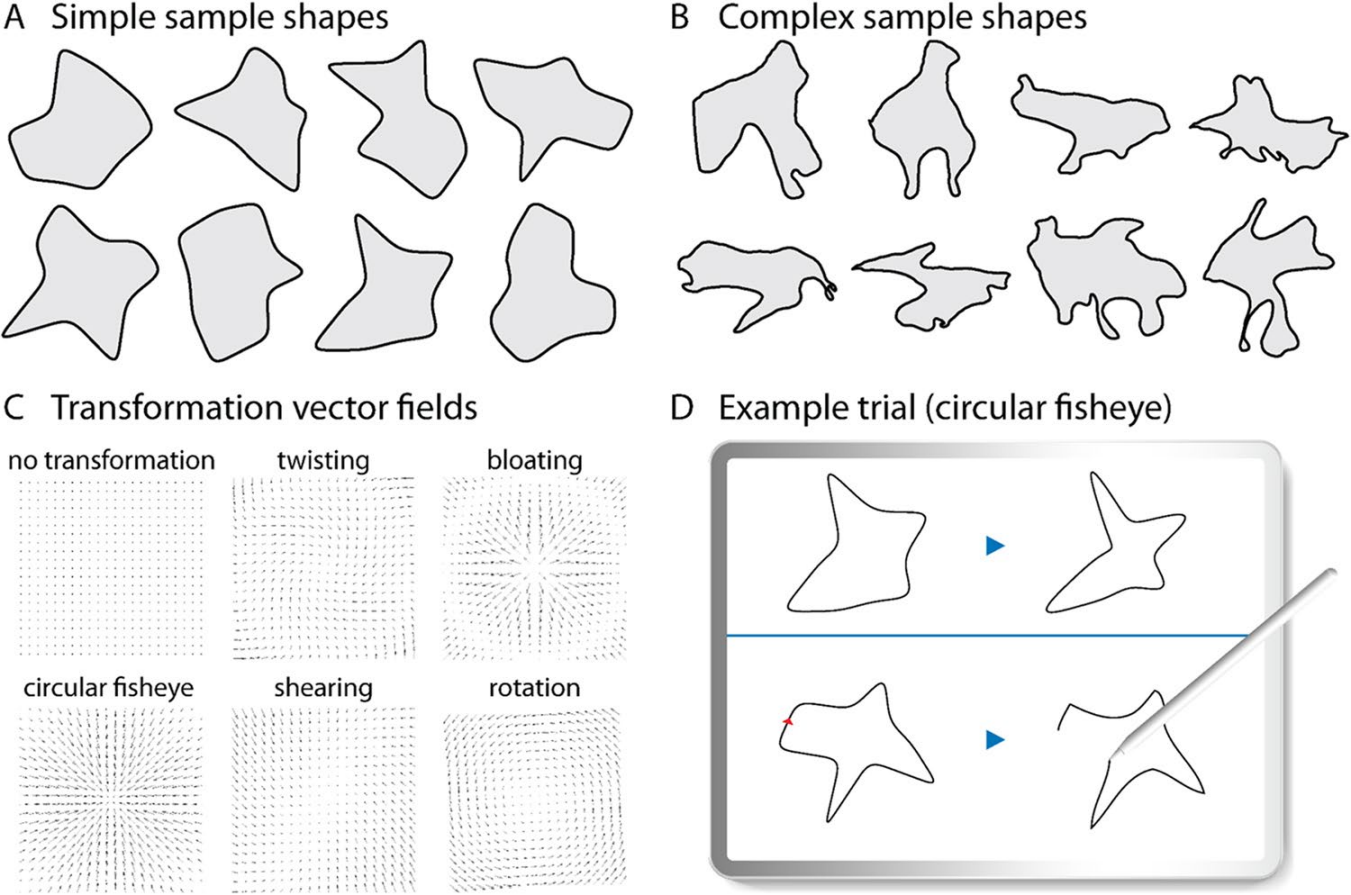
Stimuli and procedure. **A** The eight simple sample shapes. **B** The eight complex sample shapes. **C** Vector fields of the five transformations. **D **Example trial. Images of tablet and stylus are designed by rawpixel.com/Freepik

For each experiment, we created eight sample shapes (Fig. 2A, B), which we subjected to five different geometric transformations: twisting, bloating, circular fisheye projection, shearing and rotation (transformation vector fields in Fig. 2C). Before transformation, we sampled each shape uniformly and scaled to range from [0, 1] by first subtracting the absolute minimum value of each coordinate, and then dividing by the resulting absolute maximum value. We then translated the shape such that its centroid was placed at (0, 0), effectively setting the shape to a range between [−0.5, 0.5]. Except for rotation, the transformed shape was also scaled to range from [0, 1]. In addition, the twist-transformed shapes were rotated using Procrustes to better match the untransformed shape.

**Fig. 3:**
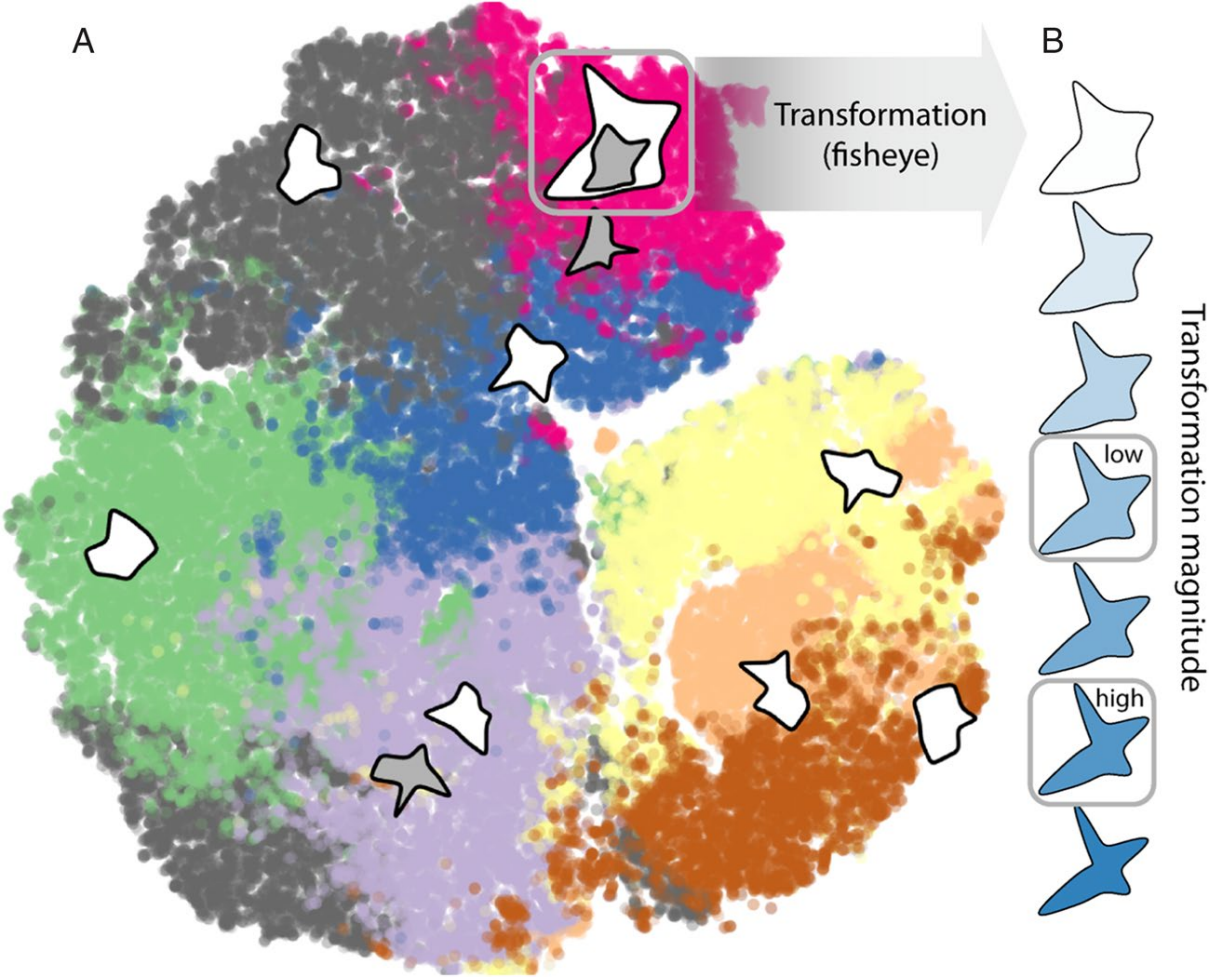
Stimulus generation illustrated for simple shapes. **A** We identified eight clusters of shapes (different colors) from 100,000 random polygons. The shapes closest to the mean of each of these clusters were our sample shapes (white). Test shapes (grey) for each sample shape were picked with low, medium, or high similarity to the sample (i.e., distance in shape space). **B** We then generated the transformed sample by transforming (here: circular fisheye projection) the sample shape with different magnitudes and selecting the transformed samples with low (0.65 ShapeComp units) and high (0.9 ShapeComp units) transformation magnitudes. (Color figure online)

We standardized transformation strength with distance in ShapeComp (ShapeComp Units), as distance in ShapeComp is predictive of human shape similarity (Fig. 5b in Morgenstern et al., 2021). Each transformation was realized with two different magnitudes: low and high (0.65 and 0.9 ShapeComp units for simple shapes; and 0.55 and 1 for complex shapes). These low and high ShapeComp transformation magnitudes led to transformed shapes that were more similar (low) or dissimilar (high) to the original test shapes. As an additional factor, similarity between sample and test shapes varied in three roughly equidistant steps: low, medium, and high (1.5, 0.5, and 0.15 for simple shapes; and 2.25, 1.5, and 0.75 for complex shapes), again adjusted by ShapeComp. Specifically, for the simple shapes we generated 100,000 random polygons (based on 10 vertices). We used *k*-means to find eight clusters of shapes based on their ShapeComp coordinates, taking the sample shape that was nearest to the mean of these clusters (Fig. 3A). We then transformed the sample shapes by varying degrees to find transformed samples that varied in similarity relative to the original sample (Fig. 3B). For each sample, we also looked for test shapes that were low, medium, or high in similarity relative to the sample (Fig. 3A). This led to 240 possible combinations of sample shapes (8), transformation magnitude (2), transformation type (5), and similarity of test to sample (3). From this set we randomly chose 60 stimuli with the condition to sample from each of the 5 transformations, 2 magnitudes, and 3 similarities at least two times. Each observer completed a drawing for each stimulus. Given there were 11 observers this led to 660 total drawings (+ 88 control drawings).

For the complex shapes, we drew shape samples from a generative adversarial network (GAN) trained to synthesize novel naturalistic shapes and then placed them within ShapeComp (Morgenstern et al., 2021). We chose to diversify sample shapes by sampling eight shapes in ShapeComp space that fell near coordinates that formed a cube in the first three dimensions of ShapeComp (where each cube vertex point fell within 1 standard deviation from ShapeComp’s origin, based on the variance of natural animal shapes). Like the simple shapes, we transformed the complex sample shapes by varying degrees, and selected test shapes that were low, medium, or high in similarity relative to the sample. This again led to 240 possibilities. Given that these shapes were more complex, to make the experiment more manageable, we asked observers to make 40 drawings (rather than 60 in the simple case). We, thus, sampled from the 240 possibilities randomly ensuring that over two groups of 10 observers (that were asked to draw a different set of 240 possibilities) we had sampled from each of the 5 transformations, 2 magnitudes, and 3 similarities at least two times (and in some cases three times). Given there were 20 observers this led to 800 total drawings (+ 160 control drawings).

### Procedure

Before the start of the experiment, participants were asked to rate their drawing skills on a 10-point Likert scale. The following drawing task was conducted on an iPad Pro (12.9-inch) in landscape orientation, with the top row showing a sample shape and the corresponding transformed sample shape, and the bottom row showing a test shape and a rectangular drawing area (about 11.2 cm^2^ in size; Fig. 2D). Participants were instructed to draw with a digital pen “how the test would look if it were to be changed in the same way as the sample shape.” A red triangle was presented on the contour of the test shape to indicate at which point and in which direction (always clockwise) participants should draw the transformed test shape. To make all drawings closed contours, participants were only able to continue drawing from the end-point of the last drawn segment. However, they could always undo the last drawn segment or clear the drawing area and start over. If participants closed the shapes (criterion <10 pixels between first and last drawn point), the drawing area turned green at which participants could either proceed to the next trial or clear the drawing area and start over.

### Data analysis

**Fig. 4:**
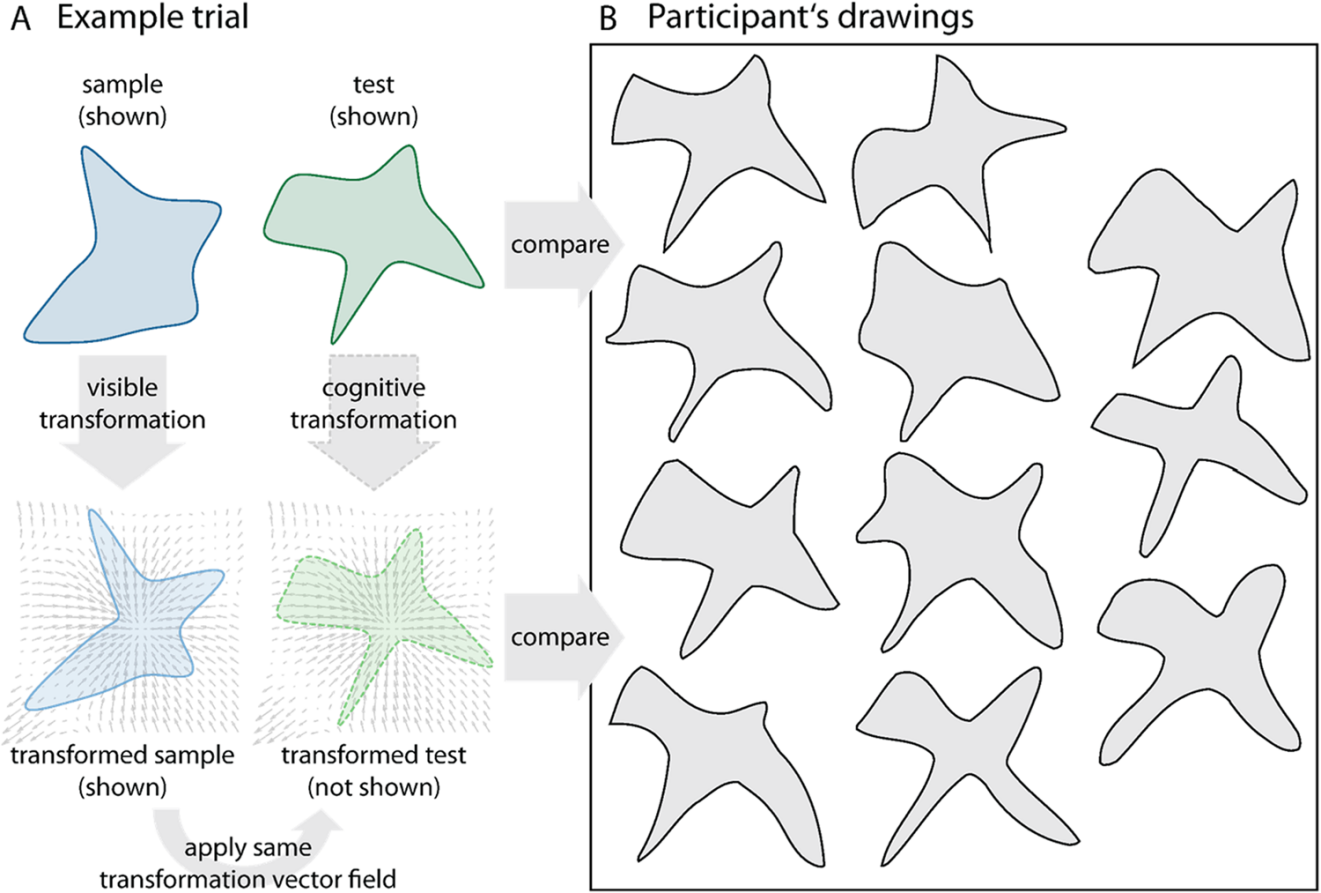
Example trial and findings. **A** Participants are presented with a sample (dark blue) and transformed sample (light blue) and asked to reproduce the transformation by applying it to the test (dark green). The transformed test shape (light green) that is used to evaluate participants’ performance is obtained by applying the vector field of the sample transformation to the test. **B** The individual drawings are compared with the test and the transformed test to see which one they are fitting better—if participants can infer and reproduce observed transformations, then their drawings should be more similar to the transformed test compared with the test. (Color figure online)

For analysis, we scaled each drawing to the range [0, 1] by first subtracting the absolute minimum value from each coordinate, and then dividing by the resulting absolute maximum value. We then translated the shape such that its centroid was placed at (0, 0), effectively setting the shape to a range between [−0.5, 0.5]. The critical comparison to evaluate participants’ performance is between each drawing and the corresponding test shape and between each drawing and the transformed test shape (Fig. 4A, B). If the drawing were more similar to the test shape it would indicate that participants reproduced the test, if the drawing were more similar to the transformed test shape it would indicate that participants were able to infer and reproduce the observed transformation. We compute these similarities by matching the center of the drawing to the center of the test shape, and to the center of the transformed test shape, and calculate the respective distances in ShapeComp space. We define similarity/dissimilarity in terms of distance in ShapeComp space, where higher distances in ShapeComp lead to more dissimilar shapes (Morgenstern et al., 2021), and, therefore, smaller distances between shapes leads to greater similarity. Then we calculate the difference between the two, *diff* = dissim(drawing to transformed test shape) – dissim(drawing to test shape), so that negative values indicate a higher dissimilarity to test shapes (i.e., more similarity to the transformed test shape), and positive values indicate higher dissimilarity to the transformed test shape (more similar to the test). This difference measure constitutes a conservative estimate of participants’ ability to infer transformations, because (i) we use unfamiliar shapes, and (ii) abstract, geometric transformations, (iii) we control for the similarity across transformations, (iv) we do not correct for individual differences in drawing skills, and (v) we use a model of human shape similarity to compare drawings not only against transformed test shapes but also against test shapes which are relatively similar.

#### *Hypotheses*

In this study, we investigate the ability of humans to infer and reproduce transformations from visual observation. Specifically, we test the following hypotheses: Participants can infer and reproduce transformations from observation, indicated by higher similarity between their drawings and transformed test shapes versus untransformed test shapes (H1). This similarity between drawings and transformed test shapes is different for different transformation types (H2A), higher for higher transformation magnitudes (H2B), higher for higher similarity between sample and test shapes (H2C), and lower for more complex stimuli (H2D).

## Results Experiment 1 (Simple shapes)

**Fig. 5:**
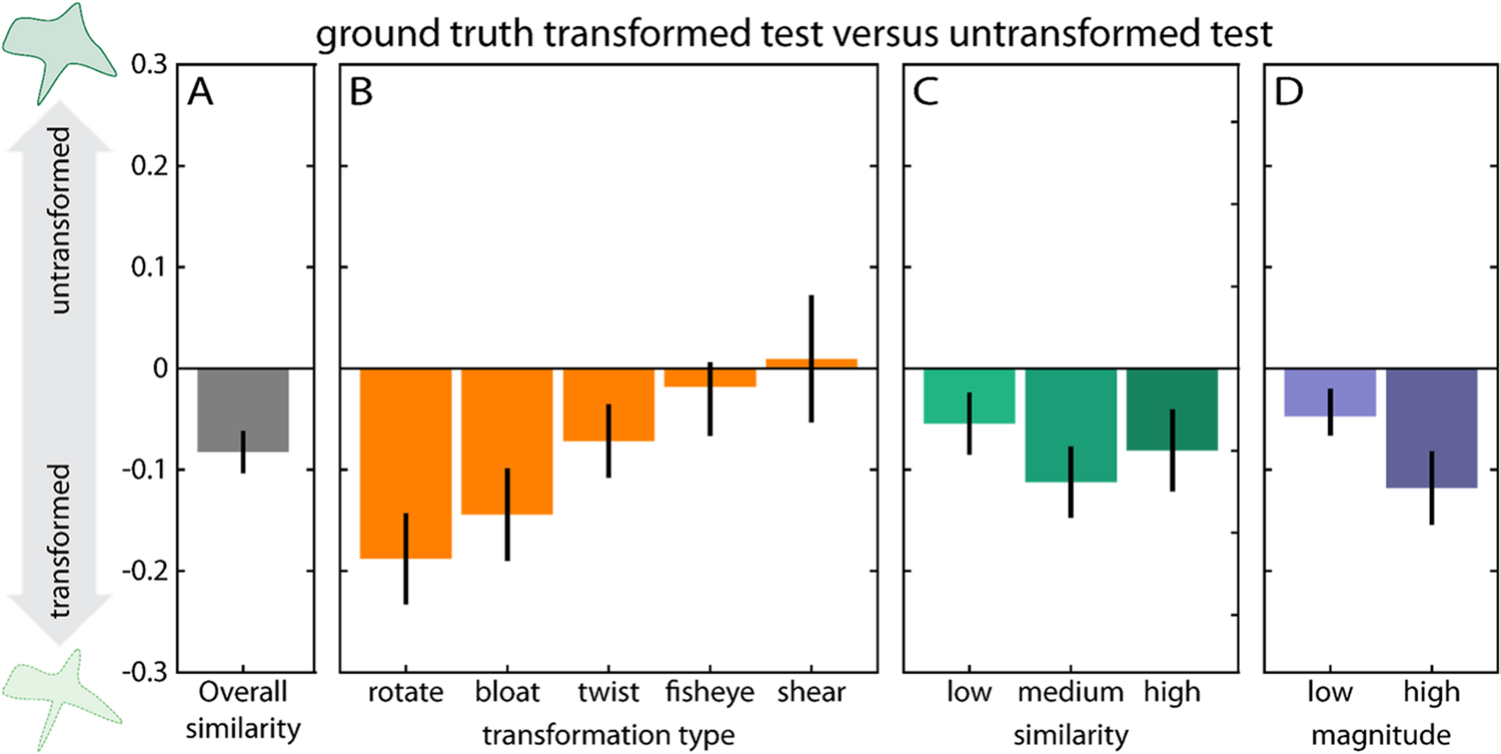
Participant performance with simple shapes, displayed as similarity to test shape (positive values) versus transformed test shape (negative values). (**A**) Overall performance. (**B-D**) Main effects of transformation type, similarity between sample and test, and transformation magnitude. Error bars denote 95% confidence intervals

Across all conditions, we find that participants’ drawings are more similar in ShapeComp space to the transformed test than the untransformed test, *t*(659) = −7.8,* p* < .001 (Fig. 5A), illustrating participants’ ability to infer and reproduce shape transformations from examples. However, we also find strong modulating effects of transformation type, *F*(4, 655) = 13.39, *p* < .001 (Fig. 5B) and transformation magnitude, *t*(329) = 3.35, *p* < .001 (Fig. 5D), with no statistically significant differences for different levels of similarity between sample and test stimulus, *F*(2, 657) = 2.52, *p* = .08 (Fig. 5C).

Specifically, when it comes to transformation type, participants are better (i.e., closer to the transformed test) at inferring and reproducing some transformations compared with others: specifically, rotation, *t*(131) = −8.20, *p* < .001, bloating, *t*(131) = −6.18, *p* < .001, and twisting, *t*(131) = −3.88, *p* < .001, were closer to the transformed compared with the untransformed test, while this was not true for fisheye, *t*(131) = −1.45, *p* = .15, and shear, *t*(131) = 0.29, *p* = .77 (Fig. 5B). We also find a main effect of transformation magnitude, with better performance for higher compared with lower transformation magnitudes, *t*(329) = 4.8, *p* < .001 (Fig. 5D). For the effects of transformation similarity and magnitude separately for the different transformation types see Suppl. Figs. S1, S2. With respect to the similarity between sample and test shape, we find better inferences and reproductions of transformed tests at high, *t*(219) = −3.90, *p* < .001, medium, *t*(219) = −6.25, *p* = .42, as well as low, *t*(219) = −3.49, *p* < .001, similarity (Fig. 5C).

## Results Experiment 2 (Complex shapes)

**Fig. 6:**
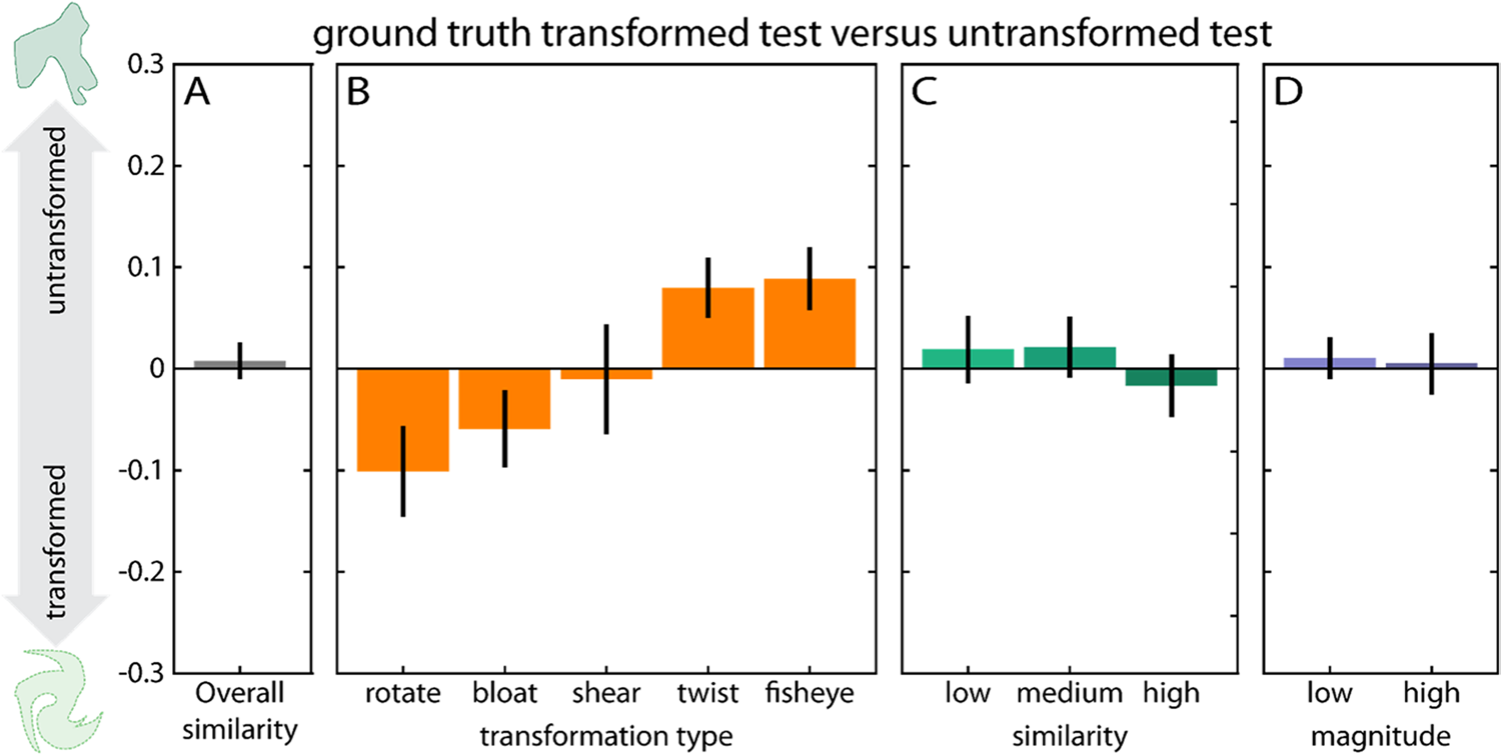
Participant performance with complex shapes, displayed as similarity to test shape (positive values) versus transformed test shape (negative values). **A** Overall performance. **B–D** Main effects of transformation type, similarity between sample and test, and transformation magnitude. Note that transformations in (**B**) are sorted by performance so the order is different compared with Fig. 5. Error bars denote 95% confidence intervals. (Color figure online)

Across all conditions, participants’ drawings are not significantly more similar in ShapeComp space to the transformed test than the untransformed test, *t*(799) = 0.83,* p* = .41 (Fig. 6A), illustrating that with complex shapes participants’ ability to infer and reproduce shape transformations is lower than with simple shapes. However, we still find strong modulating effects of transformation type, *F*(4, 795) = 17.39, *p* < .001 (Fig. 6B), even though no statistically significant effects of similarity between sample and test stimulus, *F*(2, 797) = 1.8, *p* = .17 (Fig. 6C), or transformation magnitude, *t*(389) = 0.05, *p* = .96 (Fig. 6D). Specifically, when it comes to transformation type, participants are better (i.e., closer to the transformed test) when inferring and reproducing some transformations like rotation, *t*(119) = −4.38, *p* < .001, and bloating, *t*(179) = −3.05,* p* < .001, but their drawings are more similar to the untransformed test for twisting, *t*(179) = 5.22, *p* < .001, and fisheye, *t*(179) = 5.59, *p* < .001, while in between for shear, *t*(139) = −0.37, *p* = .71. The best performance for rotation and bloating replicates our findings in simple shapes. For the effects of transformation similarity and magnitude separately for the different transformation types see Suppl. Figs. S3, S4.

## Visualizing drawings in shape space

**Fig. 7:**
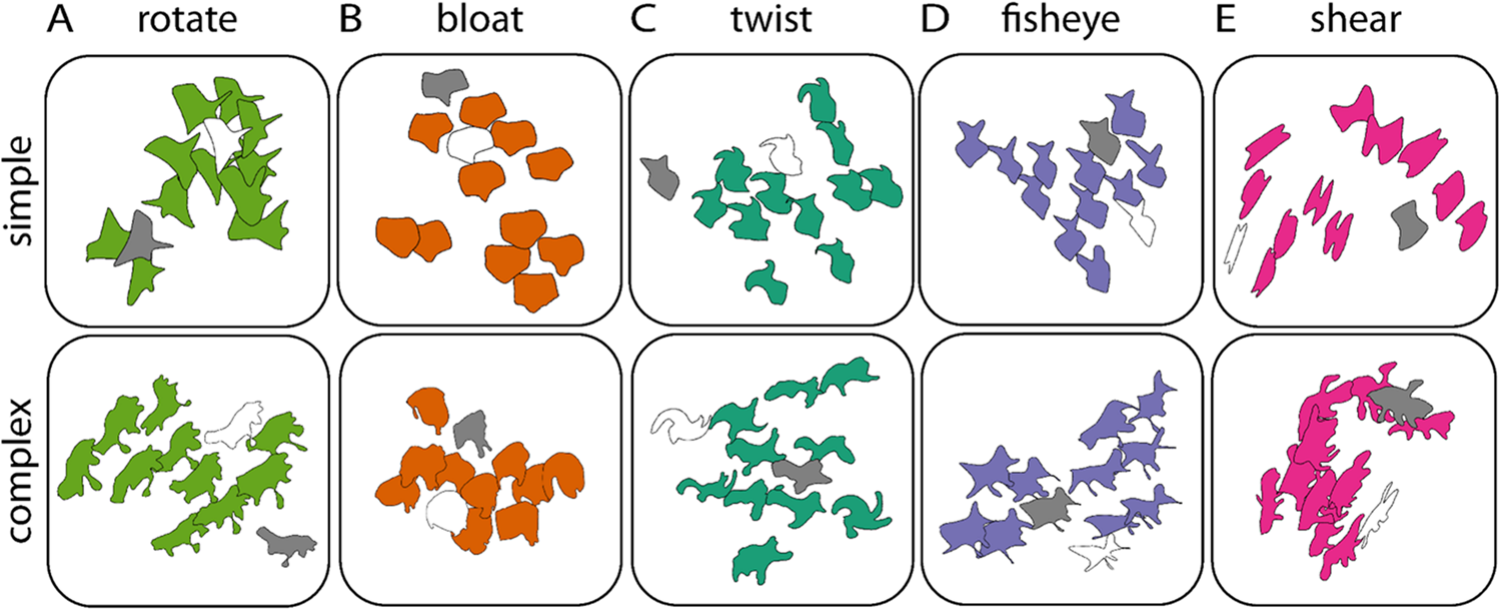
Visualizations of example drawings in shape space for high magnitude transformations. Here, we use t-Distributed Stochastic Neighbor Embedding (Van der Maaten & Hinton, 2008) to project drawings (colored) into a two-dimensional representation of ShapeComp space (Morgenstern et al., 2021). For transformation types (**A**) rotation, (**B**) bloating, and (**C**) twisting, most drawings are closer (i.e., more similar) to the transformed test (white) compared with the untransformed test (grey). This is less clear in (**D**) fisheye and (**E**) shear transformations, and more pronounced for simple (first row) compared with complex shapes (second row). (Color figure online)

Because we can project all shapes and drawings into ShapeComp space (Morgenstern et al., 2021), we can visualize the relative differences between participants’ drawings and the transformed and untransformed test shapes. If participants can infer and reproduce transformations from examples, drawings should be more similar (i.e., closer in shape space) to the transformed tests. In Fig. 7, we plot examples for simple and complex shapes and different transformation types so that we can visually evaluate the relative distances of drawings to the transformed test shapes (white) versus the untransformed test shapes (grey). This demonstrates the power of combining rich drawing data with tools from computational modeling and machine learning to explore the richness of mental representational (shape) spaces (Bainbridge, 2021).

## Discussion

Even after massive changes in object shape, such as when crumpling a towel or folding a sheet of paper, we can still identify most objects (Fig. 1). Interestingly, there is good evidence that we can not only recognize objects across such transformations but also recognize transformations across objects: We can infer growth or aging processes, crumpling, or folding from the associated changes in object shapes (e.g., Chen & Scholl, 2016; Kourtzi & Shiffrar, 1999, 2001; Mark & Todd, 1985; Pinna, 2010; Pittenger & Todd, 1983; Schmidt & Fleming, 2018; Spröte et al., 2016; Toscani et al., 2020; Yoonessi & Zaidi, 2010). One recent hypothesis about how this is achieved is “shape scission,” which assumes that observers can distinguish between shape features that “belong” to the object and those that “belong” to the transformation (Fleming & Schmidt, 2019; Phillips & Fleming, 2020; Schmidt et al., 2019; Spröte & Fleming, 2016).

Here, we put this hypothesis to the test by studying the ability of naïve participants to reproduce the effects of geometric transformations, specifically, by asking them to observe the effect of a transformation, “apply” it to a different object and draw the envisaged result. This unconstrained task is an especially conservative test of “shape scission” as (i) contour stimuli as well as transformations were novel to participants, and (ii) participants had to generate effects of transformations (instead of, for example, deciding which target is most similar to the envisaged result; e.g., Fleming & Schmidt, 2019; Schmidt et al., 2019). This task comes with particular challenges, as participants did not just have to identify and reproduce transformation features (e.g., the twirls of a twisted shape). Rather, they had to simulate its detailed effects on a different object (e.g., where a twist might or might not produce twirls). Also, even though drawing is a powerful and rich tool for measuring mental representations compared with psychophysical methods (e.g., Bainbridge et al., 2019; Hall et al., 2021; Sayim & Wagemans, 2017; Tiedemann et al., 2022), responses are far more variable than with a two-alternative forced-choice categorization task. This is a consequence of individual variability in, for example, motor abilities, backgrounds in culture or graphic systems, or drawing expertise (also our participants were no artists; e.g., Bainbridge, 2021; Chamberlain et al., 2019; Cohn, 2020; Kozbelt & Ostrofsky, 2018).

However, as a consequence of this conservative approach, any successful reproduction of a transformation on novel shapes demonstrates the ability of participants to split observed shapes into original shape and transformation. We operationalized this as higher similarity between drawings and transformed “ground truth” test—which were never shown to participants—in contrast to similarity between drawings and untransformed test. We only observed this ability for our set of simple shapes, potentially for two reasons: First, with complex shapes it is harder to envisage the transformed result—as there are more shape features to be accurately changed (e.g., more “limbs”). Second, with complex shapes it is harder to draw this result—as the noise in drawing presumably accumulates with stimulus complexity (e.g., inexperienced drawers might have difficulty in reproducing complex shapes, let alone, depicting the effects of transformation). In line with our hypotheses, we also found different effects depending on the type of geometric transformation (in simple as well as complex shapes), even though we approximately matched the perceived magnitude between transformations (using ShapeComp distance, a perceptually validated metric of perceived shape similarity). Why exactly we do observe these differences between transformation types is unclear. However, they suggest that some geometric transformations are more difficult to infer and/or reproduce than others. Most notably, we found the best performance for rotation and bloating (in simple as well as complex shapes), suggesting that those transformation types are particularly salient and easier to identify and reproduce compared with the other tested transformations.

Finally, in simple shapes, we found that transformation magnitude modulated the effect, with lower accuracy in reproducing the ground truth transformation for lower magnitudes. This effect of magnitude was broadly in line with our expectations as transformations with higher magnitudes are potentially easier to observe and therefore to reproduce—consider, for example, a steam roller that transforms every deformable object into a flat splat. In contrast to our expectations, we did not observe an effect of different levels of similarity between sample and test. We hypothesized a monotonic increase of reproduction accuracy with similarity, as higher similarity between the features of both shapes should make it easier to “translate” the transformation to the other shape. Even though low levels of similarity descriptively produced the lowest performance, this effect was not significant.

Overall, our results suggest that participants can indeed distinguish between shape features that “belong” to the object and those that “belong” to the transformation (Fleming & Schmidt, 2019; Phillips & Fleming, 2020; Schmidt et al., 2019; Spröte & Fleming, 2016)—however, this ability is subject to many factors, such as transformation type and magnitude, which can either make it easier or impossible for participants to infer transformations independently of the object. Our study also illustrates once again how we can use drawing as a tool to measure mental representations, without introducing experimenter bias by for example preselecting responses for participants to choose from. This is especially important when mapping out mental representational spaces (e.g., of object or scene categories; Bainbridge et al., 2019; Tiedemann et al., 2022).

### Limitations and future studies

Our study was specifically designed to be a conservative test of “shape scission.” Consequently, we find rather small effects and would expect much stronger results when (i) allowing participants to rely on their previous knowledge and semantic concepts by using familiar objects and transformations (e.g., reproduce dents on cans of soda), (ii) using a perceptual rather than image-computable accuracy measure (e.g., ask other participants to rate whether the transformation was faithfully reproduced), (iii) using more ecologically valid transformations (e.g., growth), or (iv) multimodal stimuli and responses (e.g., such as reproducing a particular folding of a piece of paper or twisting a chunk of clay in the real world). Finally, even though the drawing method provided us with rich data about participants’ mental representations of transformed shapes, further developments are necessary to make the most of this data: For example, we do not yet have (computational) analyses for estimating meaningful averages of individual deviations of drawings from the “ground truth” transformed test shape—from which we might learn about the general biases in the representation of transformations.

## Conclusion

We use drawings as a method to investigate “shape scission” in naïve participants. Specifically, we showed participants sample shapes before and after a geometric transformation, and asked them to reproduce the observed transformation on a novel shape by drawing the envisaged outcome. We found that for simple shapes the resulting drawings were on average more similar to the (not shown) transformed “ground truth” test than to the original test shape. However, this was not the case for complex shapes, and even in simple shapes the accuracy of reproduction was modulated by transformation type and magnitude. Together, our findings suggest that we can distinguish between representations of original object shapes and their transformations, and can use visual imagery to mentally apply nonrigid transformations to different objects. Such abilities are an important aspect of how we not only perceive but also “understand” shape. Future work should identify and characterize the factors limiting these abilities.

## Supplementary Information

Below is the link to the electronic supplementary material.

Supplementary file1 (PDF 331 KB)

## Data Availability

All stimuli and raw data (drawings) are available at https://doi.org/10.5281/zenodo.8297790, and none of the experiments was preregistered.
