# Supplementary material for: Inferring shape transformations in a drawing task
Source: Mem Cognit. 2023 Sep 5;53(1):189–99. doi: 10.3758/s13421-023-01452-0 (PMC11779755; doi:10.3758/s13421-023-01452-0)
Supplement: Supplementary file 1 — Supplementary file1 (PDF 331 KB) [file 13421_2023_1452_MOESM1_ESM.pdf]

Supplementary information

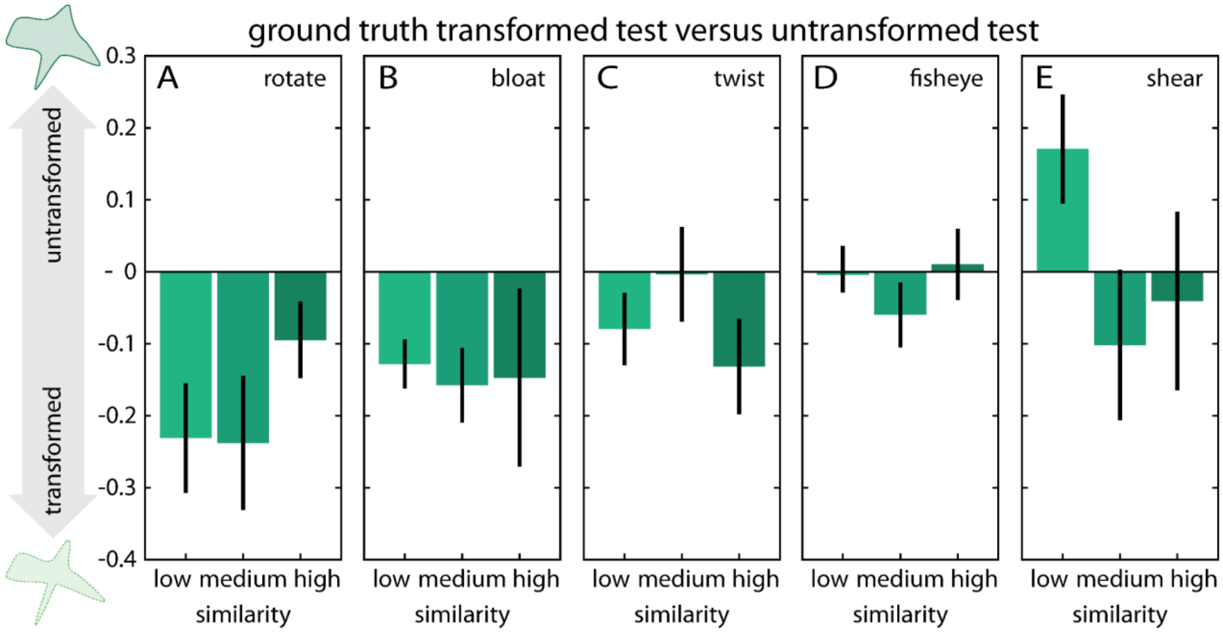

Figure S1. Effects of similarity between sample and test with simple shapes (Experiment 1), reported separately for each transformation type. The panels are ordered according to overall performance (**Fig. 5B**). Error bars denote 95% confidence intervals.

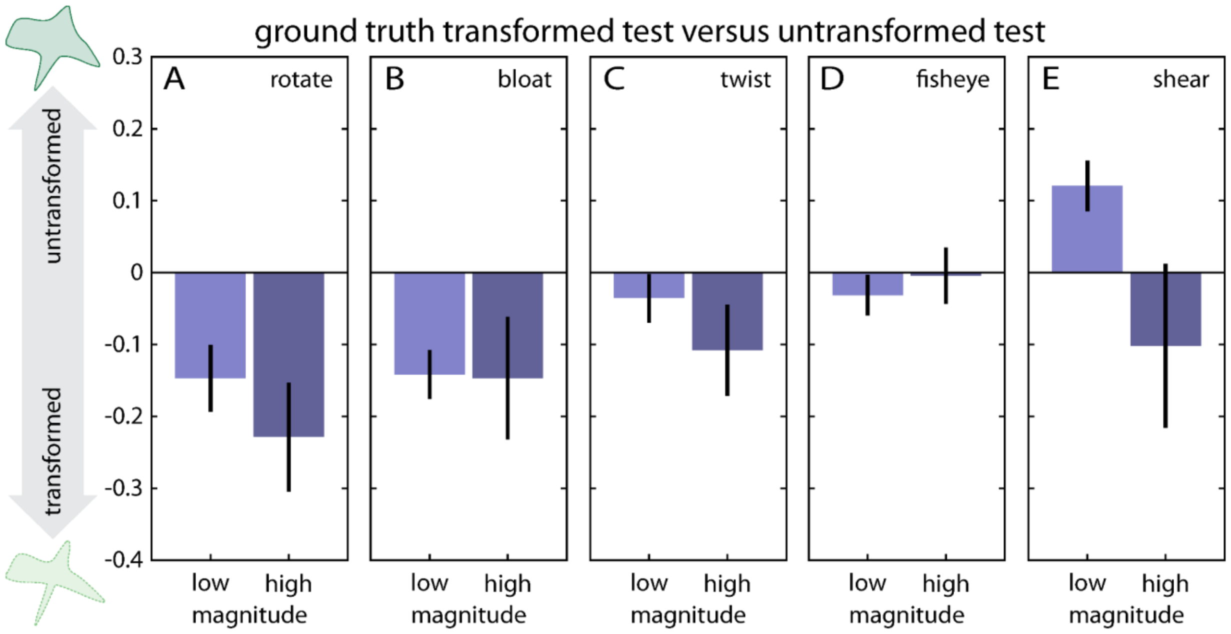

Figure S2. Effects of transformation magnitude with simple shapes (Experiment 1), reported separately for each transformation type. The panels are ordered according to overall performance (**Fig. 5B**). Error bars denote 95% confidence intervals.

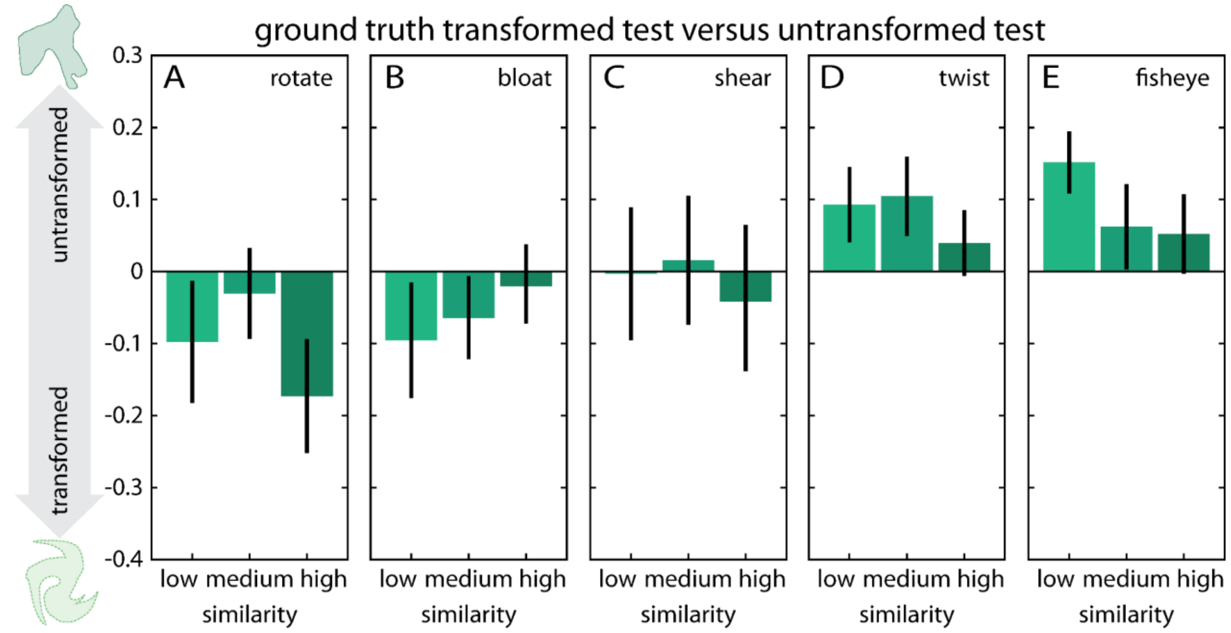

Figure S3. Effects of similarity between sample and test with complex shapes (Experiment 2), reported separately for each transformation type. For easier comparison, panels are in the same

order as in **Fig. S1**. The panels are ordered according to overall performance (**Fig. 6B**). Error bars denote 95% confidence intervals.

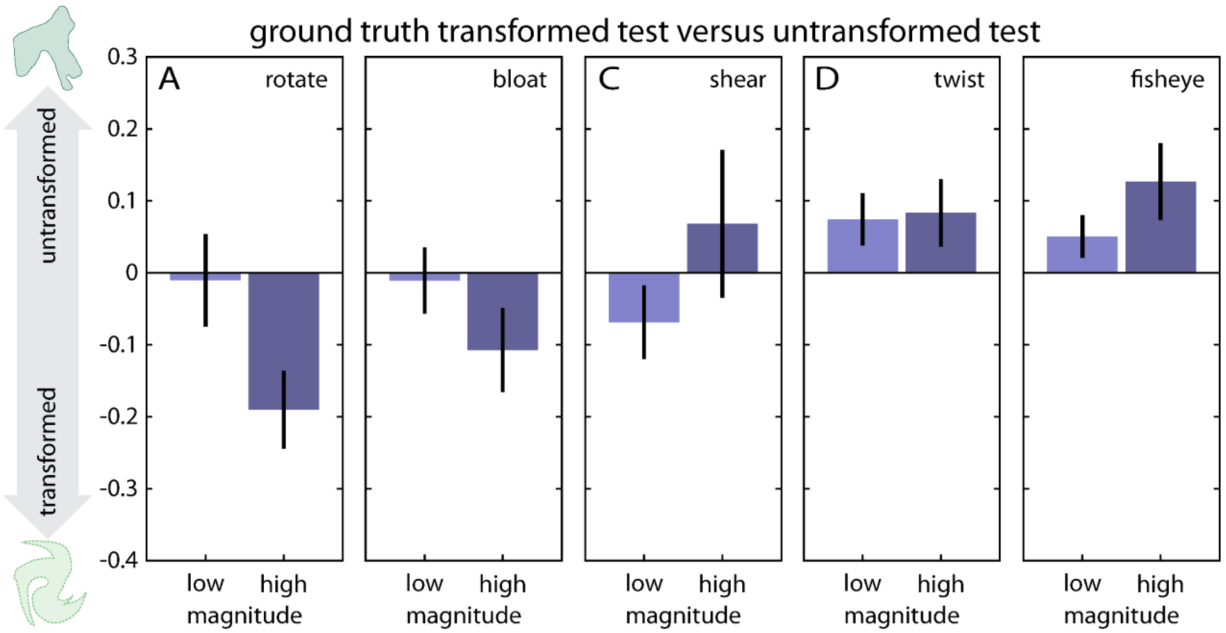

Figure S4. Effects of transformation magnitude with complex shapes (Experiment 2), reported separately for each transformation type. For easier comparison, panels are in the same order as in **Fig. S2**. The panels are ordered according to overall performance (**Fig. 6B**). Error bars denote 95% confidence intervals.
